# The influence of ligament biomechanics on proximal junctional kyphosis and failure in patients with adult spinal deformity

**DOI:** 10.1002/jsp2.1277

**Published:** 2023-08-25

**Authors:** Micah Blais, Bahar Shahidi, Brad Anderson, Eli O'Brien, Courtney Moltzen, Tina Iannacone, Robert K. Eastlack, Gregory M. Mundis

**Affiliations:** ^1^ Department of Orthopaedic Surgery Scripps Clinic Medical Group San Diego California USA; ^2^ Department of Orthopaedic Surgery UC San Diego La Jolla California USA

**Keywords:** adult spinal deformity, ligament, proximal junctional failure, proximal junctional kyphosis, spine

## Abstract

**Purpose:**

It is unknown whether the biomechanics of the posterior ligamentous complex (PLC) are impaired in individuals undergoing surgery for adult spinal deformity (ASD). Characterizing these properties may improve our understanding of proximal junctional kyphosis (PJK; defined as proximal junctional angle [PJA] of >10 deg from UIV‐1 to UIV + 2), as well as proximal junctional failure (PJF; symptomatic PJK requiring revision). The purpose of this prospective observational study is to compare biomechanical properties of the PLC in individuals with ASD who do, and do not develop PJK or PJF within 1 year of spinal fusion surgery.

**Methods:**

Intraoperative biopsies of PLC were obtained from 32 consecutive patients undergoing spinal fusions for ASD (>4 levels). Ligament peak force, tensile stress, tensile strain, and elastic modulus (EM) were measured with a materials testing system. Biomechanical properties and tissue dimensions were correlated with age, gender, BMI, vitamin D level, osteoporosis, sagittal alignment, PJA and change in PJA preoperatively, within 3 months, and at 1 year postoperatively.

**Results:**

Longer ligaments were associated with greater PJA change at 3 months (*p* = 0.04), and thinner ligaments were associated with greater PJA change at 1 year (*r* = 0.57, *p* = 0.01). Greater EM was associated with greater PJA at both 3 months and 1 year (*p* = 0.03). Five participants had a change in PJA of >10 1 year postoperatively, and three participants demonstrated PJF. EM was significantly higher in individuals who required revision surgery (*p* = 0.003), and ligament length was greater (*p* = 0.03). Preoperative sagittal alignment was not related to incidence of revision surgery (*p* > 0.10).

**Conclusions:**

The biomechanical properties of the PLC may be associated with higher risk for proximal failure. Ligaments that are longer, thinner, and less elastic are associated with higher postoperative PJA. Furthermore stiffer EM of the ligament is associated with the need for revision surgery.

## Graphical abstract

The biomechanics of the spinal posterior ligamentous complex is not well described in the presence of adult spinal deformity, and ligament health may contribute to biomechanical stressors after spinal fusion surgery. Our study found that individuals with adult spinal deformity that had longer, thinner, and stiffer spinal ligaments were more likely to develop proximal junctional kyphosis and failure after multilevel spinal fusion surgery. Future studies are needed to evaluate the impact of ligament health on surgical outcomes.

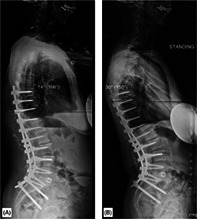

## 1 INTRODUCTION

Proximal junctional kyphosis (PJK) and proximal junctional failure (PJF) are a commonly seen outcome after instrumented fusion surgery in individuals with adult spinal deformity (ASD), with the majority of failures occurring within the first 6 months.^1^ These complications have been observed in up to 40% of individuals postoperatively, and its management is associated with significant hospital cost.^2^, ^3^ Although definitions for PJK can vary within the literature, it is typically defined as an abnormal kyphosis (typically at least 10° change in junctional sagittal Cobb angle) immediately above or at the upper instrumented vertebrae (UIV). Where PJK can present asymptomatically, it can sometimes result in clinical impairments such as recurrence of deformity, pain, and neurological deficit.^4^ In cases where PJK results in clinical impairments severe enough requiring a revision surgery, it is considered PJF. It is generally believed that the transition between the UIV and the level above it (UIV + 1) is at risk for increased biomechanical stresses due to differences in rigidity between native tissues and implants used in surgical fusion procedures. This abrupt change in rigidity manifests as disruption of the posterior ligamentous complex, vertebral fractures, instrumentation failure, degenerative disc disease, and disruption of the facet joint.^5^, ^6^

The stresses within this zone of transition are borne by the tissues supporting the junctional area, including muscle, ligament, bone, and disc. Previous investigations have examined the contributing factors of each of these tissue types, and surgeons have begun incorporating strategies to protect and augment the biomechanical properties of these tissues in an effort to reduce the risk of failure. These techniques include strategies such as UIV + 1 cement augmentation^7^, ^8^ to reduce the incidence of junctional vertebral fracture, muscle, and facet joint sparing techniques to limit damage to the posterior elements and preserve contractile strength and stability,^1^ and attention to spinopelvic alignment parameters (sagittal balance) to protect from overcorrection.^2^ Similarly, interspinous tethering^9^, ^10^, ^11^ and transverse process hooks^12^, ^13^ have gained traction to reduce risk of failure due to failure of the junctional ligamentous complex, which has been reported to be one of the more commonly observed points of failure in PJK based on imaging.^14^ Importantly, despite the frequent observation of ligamentous failure in this population, supporting data characterizing ligament health as an independent risk factor for PJK is unavailable. Prior studies investigating tissue‐specific risk factors for PJK/PJF report muscle atrophy^15^ and poor bone mineral density^16^ as independently associated with failure; however, to our knowledge no prior studies investigated the mechanical properties of the ligamentous complex as an independent risk factor. Current surgical recommendations have been based off computer simulations of cadaveric data, with no in vivo validation.^17^

With the growing incorporation of ligament augmentation techniques in surgical management of ASD, the importance of validating the underlying biomechanical and biological assumptions of ligamentous mechanical properties is paramount. The goal of our study was to determine if certain ligamentous biomechanical properties could be associated with the in vivo development of PJK/PJF. If such an association could be established, this would subsequently allow for further investigation into radiographic and biochemical characteristics of these tissues harvested from ASD patients at the time of surgery (both index and revision procedures). This would potentially allow surgeons to tailor specific treatments or preventative strategies for patients who could be identified as being “at risk” for PJK/PJF due to ligamentous concerns, with the ultimate goal of decreasing failure risk and improving patient outcomes.

## 2 MATERIALS AND METHODS

### 2.1 Participants

All participants were recruited from a single center and presented with a diagnosis of ASD eligible for either a primary or revision posterior spinal fusion surgery. ASD was defined as a patient who presented with any combination of the following characteristics: (1) a coronal cobb angle of >30°, (2) a pelvic incidence‐lumbar lordosis mismatch of >10°, (3) a sagittal vertical axis of >5 cm, or (4) thoracic kyphosis of >60°. All participants provided informed consent to the procedures indicated as approved by the relevant Institutional Review Boards (Scripps IRB 136297, UCSD IRB 805663) and in accordance with the Declaration of Helsinki. Intraoperative biopsies of the posterior spinal ligamentous complex (PLC) were obtained from individuals with a diagnosis of ASD undergoing multilevel open spinal fusions (>4 levels). Patients with no diagnosis of ASD or who were inappropriate to undergo surgery were excluded. Patients with history of previous surgical intervention at the levels of interest where the PLC was not well preserved were also excluded. Intraoperative biopsies of the PLC were harvested two levels below the upper instrumented level. PJK was defined by the following criteria based on pre‐ and postsurgical lateral radiographs^5^:

1. A proximal junctional sagittal Cobb angle of >10° *and/or*
2. At least 10° greater junctional sagittal Cobb angle as compared to a preoperative measurement.

**FIGURE 1:**
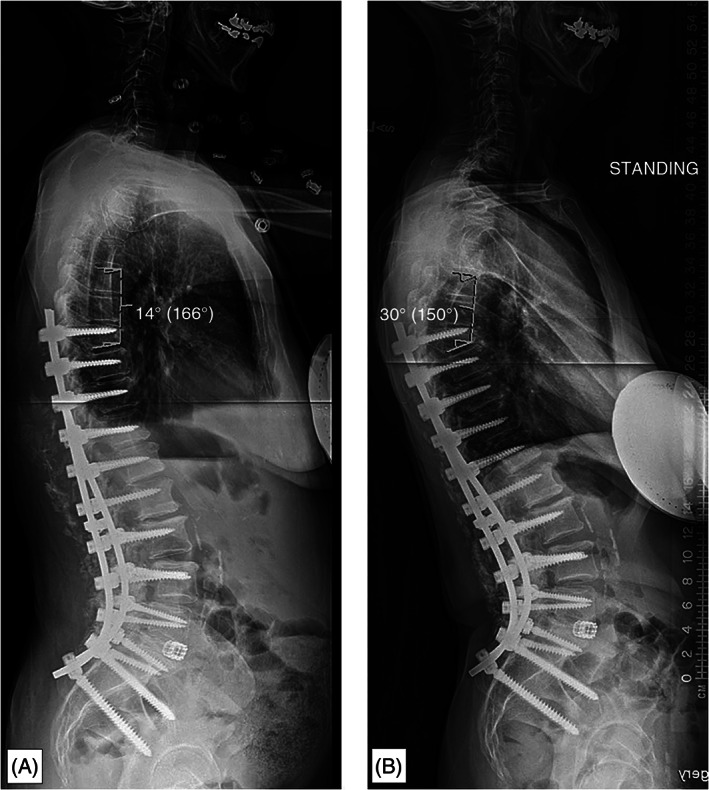
Radiographs illustrating (A) the technique used for measurement of proximal junctional angle (PJA) as well as (B) the same patient who went on to develop a 16° progression of the PJA, therefore meeting criteria as having developed proximal junctional kyphosis (PJK).^5^

Patients were categorized as having developed PJF if they developed symptomatic PJK that required surgical treatment, including revision. Participant characteristics were collected including age, gender, BMI, number of fused vertebrae, preoperative vitamin D levels, preoperative DEXA Z‐scores, parathyroid hormone levels (PTH) as indicators of overall bone health, and pre‐ and postoperative radiographic measures of sagittal alignment including sagittal vertical axis (SVA), pelvic tilt (PT), pelvic incidence‐lumbar lordosis mismatch (PI‐LL), and proximal junctional angle (PJA; Figure 1). Radiographic measurements were obtained for all patients at three different time points: preoperatively, 3‐months postoperative, and at 1‐year postoperative.

### 2.2 Mechanical properties and testing

**FIGURE 2:**
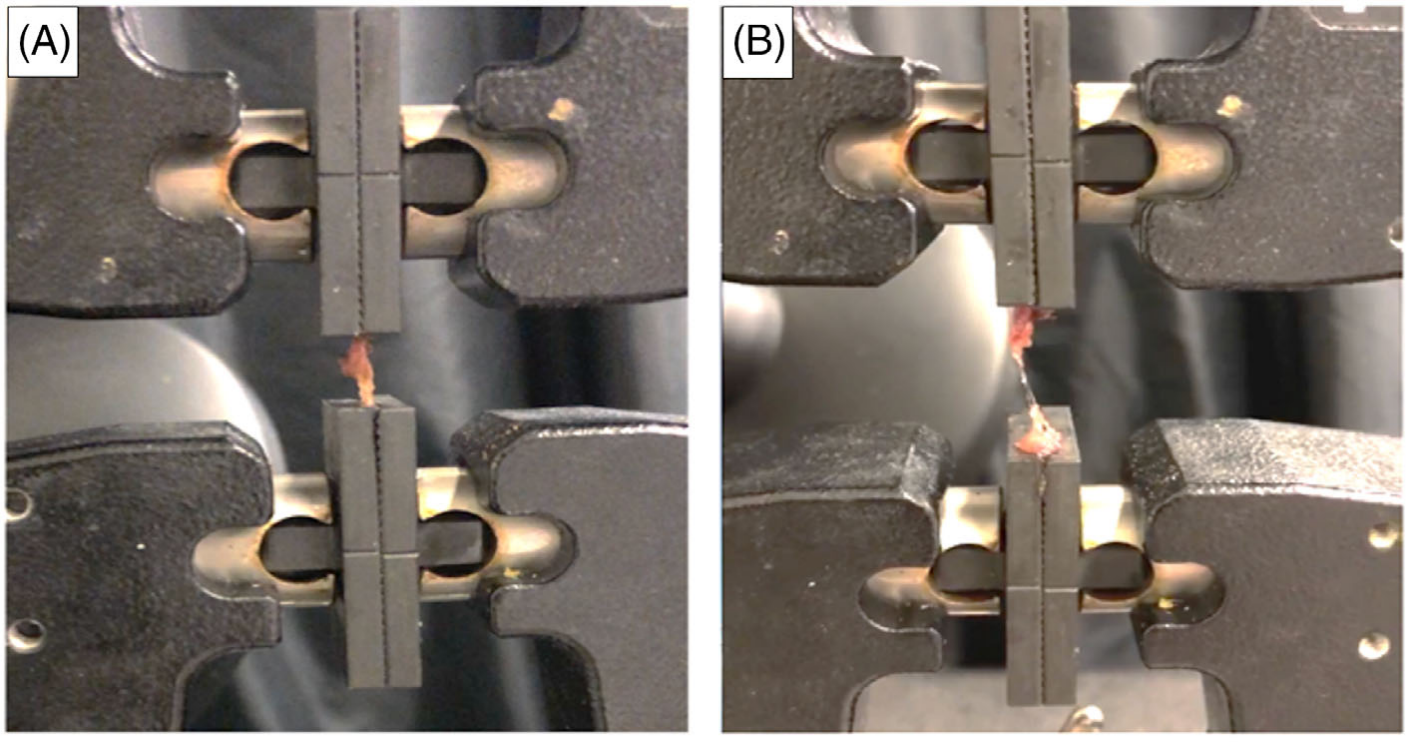
Example of biomechanical testing (A) ligament specimen before loading and (B) ligament specimen after load to failure completed.

**FIGURE 3:**
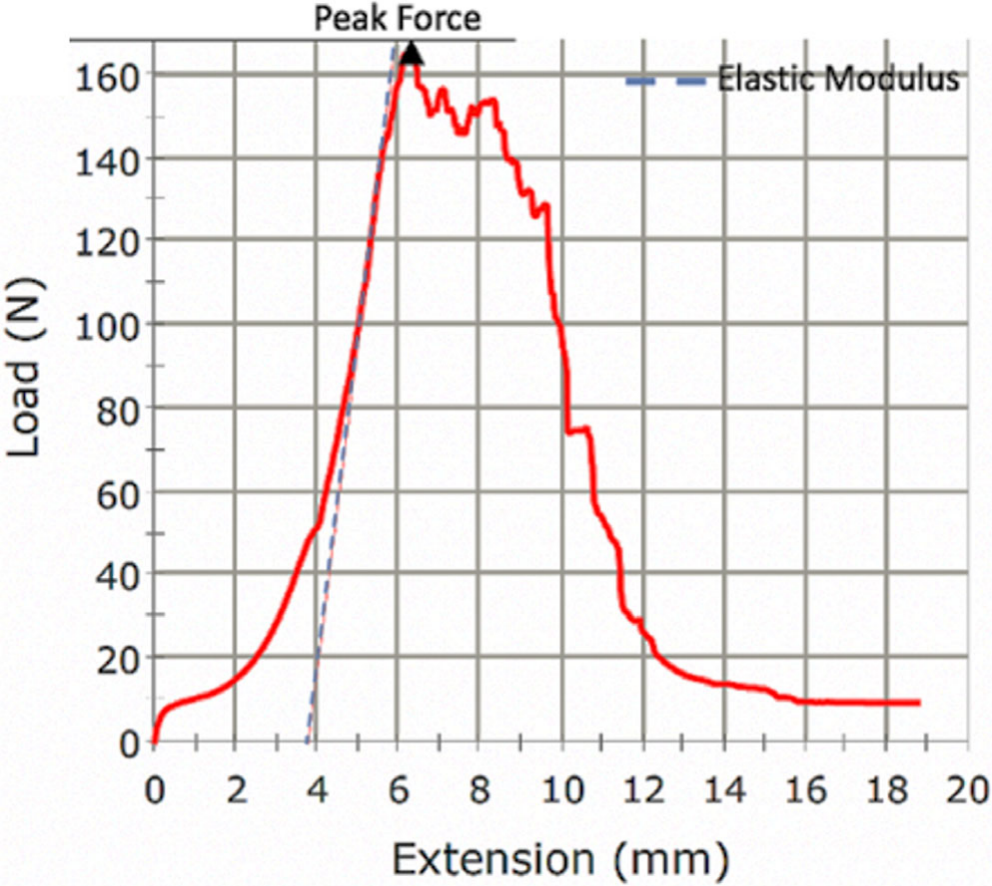
Example of a load‐strain curve generated for a ligament specimen using the materials testing system.

Intraoperative biopsies of posterior spinal ligaments (interspinous and supraspinous ligaments together) attached to their bony insertions (bone‐ligament‐bone complex) were removed for spinal fusion access with patient consent. Samples were then immediately flash frozen and stored at −80° until biomechanical testing. At the time of testing, samples were thawed to room temperature and the interspinous and supraspinous ligaments were tested in a combined manner due to (1) the inability to distinguish the interspinous and supraspinous ligaments anatomically, and (2) the fact that they do not operate in isolation for resisting tensile force in their natural environment (Figure 2). Biomechanical properties were assessed using a materials testing system (Instron Model‐5565A) to measure peak force (a measure of ligament strength), tensile stress (a measure of internal force per unit area), tensile strain (a measure of deformation), and elastic modulus (a measure of stiffness; Figure 3).

### 2.3 Statistical analysis

Statistical comparisons for patient characteristics, including preoperative and postoperative radiographic alignment parameters, as well as the biomechanical properties of the ligament specimens were performed between the patients who developed PJF and those who did not develop PJF using independent student *t*‐tests with significance set to a *p*‐value of <0.05. Patient characteristics, including preoperative and postoperative radiographic alignment parameters as well as clinical outcomes, were also correlated with the biomechanical properties and characteristics of the ligament specimens using Pearson correlation coefficients. Dimensions of the ligament, including length and thickness, were measured using calipers that were validated using microCT.

## 3 RESULTS

Of 32 patients with ASD who met the inclusion criteria, three (10.7%) developed PJF by 1‐year postoperatively. Demographics including age, gender, BMI, DEXA score, Vitamin D levels, and PTH levels are reported in Table 1. The majority (54%) of patients had lumbar‐only curves, with marked deformity (PI‐LL mismatch >20°; 52%) in the sagittal plane, moderate or marked global alignment impairment (SVA >4 cm; 54%), and moderate to marked pelvic tilt (>20°; 64%). Individual Schwabb classification designations can be found in Table S1. Five patients were found to have a PJA increase of greater than 10° between preoperative and 1‐year postoperative imaging. The mean change in PJA for the entire cohort, when compared to preoperative measurement, was 6.0 (10.2) degrees at 3‐month postoperative and 6.2 (9.8) degrees at the 1‐year postoperative time point with a mean change in PJA of 0.6 (3.6) degrees between 3‐month postoperative and 1‐year postoperative (Table 2). The amount of change in PJA did not correlate to the degree of preoperative sagittal malalignment, nor was there any correlation between the degree of sagittal correction and the need for subsequent revision surgery (*p* > 0.10).

**TABLE 1:** Demographics. Values are reported as mean (SD) by PJF status at 1‐year postoperatively. Gender is reported as a percent ratio. Significant *p*‐values (*p* < 0.05) are denoted by an asterisk.

	Total	PJF status	
		PJF	No PJF
*n*	32	3	29
Age (years)	62.8 (17.5)	76.7 (4.0)	61.3 (17.3)*
Gender (M: F)	25%:75%	33%:67%	33%:67%
BMI	25.6 (4.9)	23.1 (1.7)	26.6 (4.8)
DEXA Score	−1.9 (0.9)	−2.5 (1.2)	−1.9 (0.9)
Vit. D (ng/mL)	38.6 (12.1)	33.1 (2.8)	39.5 (12.8)
PTH (ng/mL)	62.6 (40.1)	54.2 (6.3)	64.7 (44.9)

Abbreviations: PJF, proximal junctional failure; PTH, parathyroid hormone.

**TABLE 2:** Alignment measurements for spinal parameters measured preoperatively, 3‐month postoperatively, and 1‐year postoperatively reported as mean (SD). Statistical comparisons were performed between each time point within each measurement category. Significant *p*‐values (*p* < 0.05).

	Preoperative	3‐months postoperative	1‐year postoperative
PJA (degrees°)	8.4 (7.8)	14.6 (11.0)	13.7 (10.3)
SVA (mm)	58.7 (56.3)	19.7 (32.6)^a^	9.7 (29.6)^b^
PT (degrees°)	24.9 (10.8)	21.9 (8.4)	20.5 (7.7)
PI (degrees°)	54.6 (14.9)	57.1 (13.8)	45.1 (41.2)
LL (degrees°)	−30.2 (27.1)	−47.4 (20.9)^a^	−49.2 (15.3)^b^
PI‐LL mismatch (degrees°)	13.5 (34.9)	7.1 (10.6)	−3.1 (38.8)^b^ ^,^ ^c^

Abbreviations: PI‐LL, pelvic incidence‐lumbar lordosis; PJA, proximal junctional angle; PT, pelvic tilt; SVA, sagittal vertical axis.

^a^ Adjusted *p* < 0.01 between preoperative and 3‐months postoperative.

^b^ Adjusted *p* < 0.01 between preoperative and 1‐year postoperative.

^c^ Adjusted *p* < 0.01 between 3‐months and 1‐year postoperative.

**FIGURE 4:**
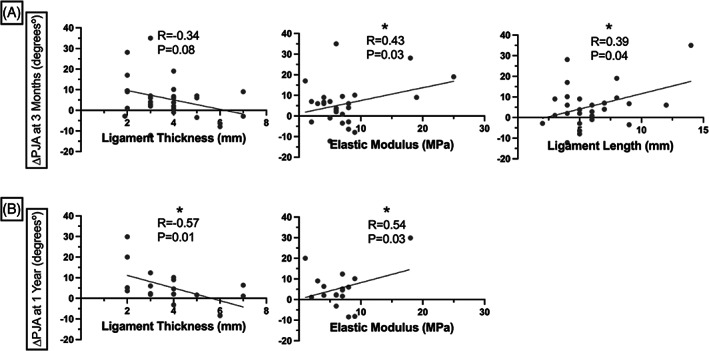
Correlations between ligament length, thickness, and elastic modulus and change in PJA between preoperative and (A) 3‐months postoperative and (B) 1‐year postoperative. Significant *p*‐values (*p* < 0.05) are denoted by an asterisk.

**FIGURE 5:**
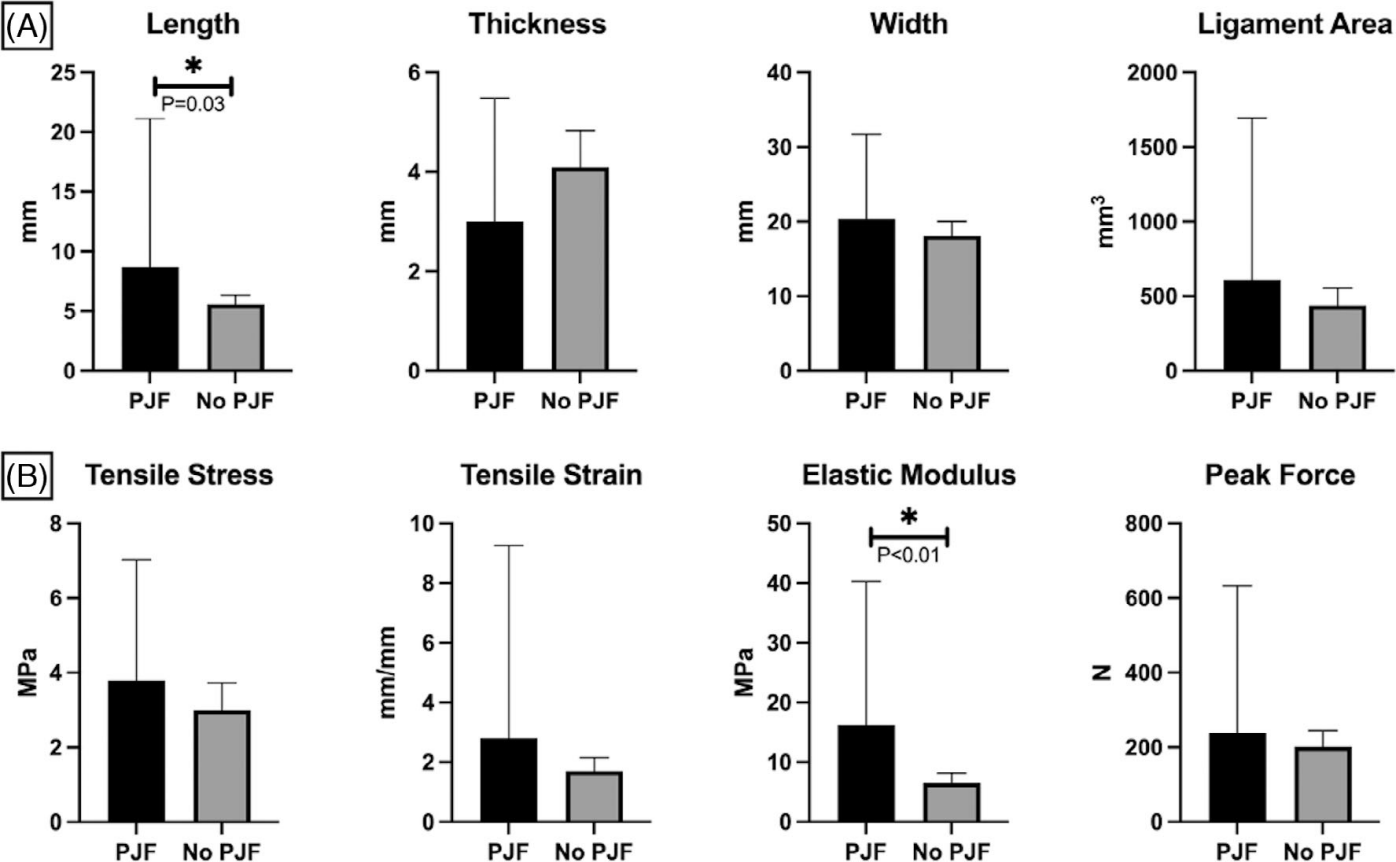
Comparison of (A) physical characteristics of ligament, and (B) biomechanical outcomes between patients who developed PJF by 1‐year and patients who did not develop PJF by 1‐year postoperatively. Significant *p*‐values (*p* < 0.05) are denoted by an asterisk.

Relative to baseline sagittal alignment parameters, we observed a significant relationship between lower ligament thickness and higher PI‐LL mismatch (*r* = −0.42, *p* = 0.02), and trends for associations between higher tensile stress and higher PJA (*r* = 0.34, *p* = 0.06), higher elastic modulus and higher PI (*r* = 0.35, *p* = 0.06), lower maximum load and higher PI‐LL mismatch (*r* = −0.34, *p* = 0.06). The physical characteristics of the ligament (Table 3) were found to correlate with the magnitude of change of PJA between pre‐ and postoperative measurements. Specifically, increased ligamentous length was found to correlate with greater change in PJA at the 3‐month time point (*r* = 0.39, *p* = 0.04; Figure 4). Additionally, decreased ligamentous thickness was also found to correlate with a greater change in PJA at both 3 months (*r* = 0.34, *p* = 0.08) and 1 year (*r* = 0.57, *p* = 0.01) postoperative time points (Figure 4). On evaluation of the biomechanical properties of the ligament specimens, increased elastic modulus (stiffness) was found to correlate positively with increased PJA at both the 3 month (*r* = 0.43, *p* = 0.03) and 1 year (*r* = 0.54, *p* = 0.03) postoperative time points (Figure 4). Increased ligament elastic modulus and ligament length were also found to be higher in individuals who developed PJF requiring revision (16.2 [9.7] MPa; 8.7 [5.0] mm) versus those who did not (6.5 [3.6] MPa [*p* = 0.003]; 5.6 [1.7] mm [*p* = 0.03]; Figure 5, Table 3).

**TABLE 3:** Physical characteristics of ligament and primary biomechanical outcomes reported as mean (SD) by PJK status at 1‐year postoperatively. Statistical comparisons were performed within groups, and significant *p*‐values (*p* < 0.05) are denoted by an asterisk.

	Total	PJK status	
		PJK (*n* = 3)	No PJK (*n* = 2)
Length (mm)	5.9 (2.6)	8.7 (5.0)	5.6 (1.7)*
Thickness (mm)	3.8 (1.5)	3.0 (1.0)	4.1 (1.6)
Width (mm)	17.7 (4.7)	20.3 (4.6)	18.1 (4.2)
Area (mm^3^)	440.8 (296.6)	607.3 (437.4)	438.6 (252.9)
Tensile stress (Mpa)	3.3 (1.5)	3.8 (1.3)	3.0 (1.6)
Tensile strain (mm/mm)	1.9 (1.2)	2.8 (2.6)	1.7 (1.0)
Elastic modulus (Mpa)	7.1 (5.1)	16.2 (9.7)	6.5 (3.6)*
Peak force (N)	207.9 (100.6)	238.4 (158.5)	201.9 (94.0)

Abbreviation: PJK, proximal junctional kyphosis.

## 4 DISCUSSION

To our knowledge, our study is the first to evaluate the relationship between biomechanical properties of the PLC patient radiographic alignment parameters and PJK or PJF. Our results indicate that ligament dimensions and elastic modulus, or stiffness, are correlated with PJA at both the 3 month and 1 year time frame. Ligamentous complex stiffness and ligament length were also found to be higher in patients who underwent revision surgery than those who did not.

The clinical significance of the PLC has been well established, particularly within the realm of spine trauma treatment. Specifically, the integrity of the PLC has been incorporated into surgical decision making models for both cervical and thoracolumbar fractures.^18^, ^19^ Furthermore, a loss of integrity of the PLC has also been demonstrated to have clinical significance for trauma patients as risk factor for neurologic injury.^20^ While these studies demonstrate the importance of the PLC in spinal trauma patients its role in ASD patients, specifically those with PJK/PJF, is not as well described within the existing literature.

Other anatomic structures potentially contributing to the development of PJK have also been more thoroughly examined by previous investigators. Decreased dimensions of the paraspinal musculature at the upper levels of instrumentation has been shown to be associated with an increased risk of PJK.^21^ Decreased bone mineral density has similarly been implicated in patients undergoing long fusion for ASD.^22^ Although we did not observe relationships between the presence of osteoporosis or other indicators of poor bony health (DEXA score, vitamin D, PTH levels) and risk of failure in our cohort, recent evidence supports the notion that the presence of osteoporosis, or the observation of lower Hounsfield Units as measured by computed tomography at the UIV, can provide important prognostic information on risk for failure.^23^, ^24^, ^25^ The role of the ligamentous complex in the development of PJK is less clear.

Prior studies investigating the contribution of biomechanical properties of ligament to injury risk are sparse, particularly in the spinal region. Multiple studies have focused on the dimensions and biomechanical properties of different graft options used in anterior cruciate ligament (ACL) reconstruction.^26^, ^27^, ^28^ These authors have demonstrated a strong relationship between the dimensions of the material used to replace the torn ligament and clinical outcomes, including graft failure/re‐rupture. In contrast, the spinal region is not well‐studied with regards to the clinical significance of the dimensions of the surrounding ligamentous structures or their mechanical properties. Kotani et al. performed an animal study showing changes in biomechanical properties of the PLC after a single level fusion. This study demonstrated lower ligament stiffness and tensile strength (>50% reduction) postoperatively at the operative level. Notably, this study was limited to a single level fusion construct and did not examine the tissue above a multi‐level fusion construct for ASD patients.^29^ Using a multi‐level ovine model, Korkmaz et al. concluded that the PLC dysfunction played a more significant role in the development of PJK than facet joint incompetency.^17^ Our study demonstrated that patients with stiffer, longer, and thinner ligament were more at risk for PJA and failure. This is contrary to the aforementioned literature but is similar to studies demonstrating that ligament morphometry is an indicator of injury risk and functional recovery. One possible explanation for the discrepancy in our findings from prior literature in the spine is that the surgical procedure and biopsy location are different. Given that prior literature examined tissue from a single level fusion at the fused level, it is reasonable to expect that the ligament at that region would experience stress shielding and exhibit reduced stiffness. In contrast, the location of biopsy from our revision cases was *above* the fused segments, which may have resulted in a need for tissue adaptation to accommodate increased biomechanical stressors. It should be noted that individuals with prior revision surgery may be at higher risk for developing PJK or PJF because of prior surgery, and the inclusion of these individuals in the current analysis may introduce an additional confounder as it relates to the secondary impact on the ligamentous complex. Future research is needed to elucidate these influences in a larger cohort.

Another reason for the contrasting findings is that the causes for PJK is likely complex, and the underlying contribution of a given tissue or anatomical structure to an individual patient's risk is variable. Indeed, many studies have attempted to explore the interrelationship between ligament mechanics and the state of the surrounding tissues. Of the handful of studies investigating spinal ligament mechanics in humans, most are performed on cadaveric samples^30^, ^31^, ^32^, ^33^ and are primarily descriptive. Other studies attempting to examine the respective roles and contributions of the various structures within a spinal segment have reached differing and sometimes contradictory conclusions. Some authors of human cadaveric studies have concluded that the mechanical properties are influenced by facet, but not disc degeneration, and decline with age.^34^, ^35^ Of note, these studies have not included patients with ASD, which limits the applicability of their conclusions to the diagnosis of PJK. Kim et al. evaluated the impact of PLC transection on the range of motion at the proximal junctional segment when compared to native and instrumented cadaveric spines.^36^ They concluded that the posterior ligamentous complex plays a less significant role in the prevention of PJK and is secondary to that of the anterior column structures. These data leave much to be explored relative to the influence of tissue health on PJK and PJF risk in vivo.

To our knowledge, there are no studies evaluating the biomechanical properties of PLC structures in patients undergoing surgery for ASD. Future research with larger sample sizes are required to further investigate the mechanisms of the interactions between tissue health and adaptability, surgically induced biomechanical stresses, and risk for injury in the spine. Additionally, radiographic correlates to tissue health biomarkers known to impact outcomes would allow for a non‐invasive way to identify at‐risk patients and adjust surgical strategies selectively.

## 5 CONCLUSION

Certain biomechanical properties of the posterior ligamentous complex correlate with increases in postoperative PJA postoperatively. Specifically, increased ligamentous complex elastic modulus (stiffness) was found to correlate with increased PJA at both the 3‐month and 1‐year postoperative time points in patients undergoing multi‐level spinal fusion for ASD. Patients in this cohort who required subsequent revision surgery were also found to have statistically significant increase in ligamentous stiffness (i.e., increased elastic modulus) and ligament length compared to those who did not require revision surgery.

## CONFLICT OF INTEREST STATEMENT

Author Bahar Shahidi is a consultant for San Diego Spine Foundation. Gregory M. Mundis is a consultant for Carlsmed, Nuvasive, Seaspine, SIBone, and Viseon; holds stock/stock options in Alphatec, Nuvasive, and Orthofix Inc; receives IP royalties from Nuvasive, Seaspine, and Stryker. Robert K. Eastlack is a consultant for Aesculap, Biedermann‐Motech, Johnson & Johnson, Medtronic, Neo Medical NuVasive, SeaSpine, & SIBone; holds stock in Alphatec, NuVasive, SeaSpine, SIBone, and Spine Innovations. These disclosures/relationships did not influence this research, and the authors have no conflicts.

## Supporting information


**TABLE S1.** Patient specific Schwabb classifications. Coronal curve types are defined as T = Thoracic only (lumbar curve <30°), L = Lumbar only (thoracic curve <30°), D = double curve (thoracic and lumbar curves >30°), or N = no major coronal deformity (all coronal curves <30°). Sagittal modifiers include PI‐LL mismatch (0 = nonpathologic, PILL<10°; + = moderate deformity, 10 < PI‐LL < 20°; or ++ = marked deformity, >20°), SVA modifiers (0 = non‐pathologic, SVA <4 cm; + = moderate deformity, 4 < SVA <9.5 cm, ++ = marked deformity, SVA >9.5 cm), and Pelvic Tilt modifiers (0 = nonpathologic, PT < 20°; + = moderate deformity 20 < PT < 30°; ++ = marked deformity, PT > 30°).
