# Supplementary material for: The influence of ligament biomechanics on proximal junctional kyphosis and failure in patients with adult spinal deformity
Source: JOR Spine. 2023 Aug 25;6(3):e1277. doi: 10.1002/jsp2.1277 (PMC10540824; doi:10.1002/jsp2.1277)
Supplement: Supplementary file 1 — TABLE S1. Patient specific Schwabb classifications. Coronal curve types are defined as T = Thoracic only (lumbar curve <30°), L = Lumbar only (thoracic curve <30°), D = double curve (thoracic and lumbar curves >30°), or N = no major coronal deformity (all coronal curves <30°). Sagittal modifiers include PI‐LL mismatch (0 = nonpathologic, PILL<10°; + = moderate deformity, 10 < PI‐LL < 20°; or ++ = marked deformity, >20°), SVA modifiers (0 = non‐pathologic, SVA <4 cm; + = moderate deformity, 4 < SVA <9.5 cm, ++ = marked deformity, SVA >9.5 cm), and Pelvic Tilt modifiers (0 = nonpathologic, PT < 20°; + = moderate deformity 20 < PT < 30°; ++ = marked deformity, PT > 30°). [file JSP2-6-e1277-s001.docx]

Supplementary Table 1. Patient specific Schwabb classifications. Coronal curve types are defined as T=Thoracic only (lumbar curve <30 degrees), L=Lumbar only (thoracic curve <30 degrees), D= double curve (thoracic and lumbar curves >30 degrees), or N=no major coronal deformity (all coronal curves <30 degrees). Sagittal modifiers include PI-LL mismatch (0=nonpathologic, PILL<10 degrees; +=moderate deformity, 10<PI-LL<20 degrees; or ++=marked deformity, >20 degrees), SVA modifiers (0=non-pathologic, SVA<4cm; +=moderate deformity, 4<SVA<9.5cm, ++=marked deformity, SVA>9.5cm), and Pelvic Tilt modifiers (0=nonpathologic, PT<20degrees; +=moderate deformity 20<PT<30 degrees; ++=marked deformity, PT>30 degrees).

| Age | Sex | Coronal | PI-LL Mod | SVA Mod | PT Mod |
| --- | --- | --- | --- | --- | --- |
| 70 | M | N | ++ | ++ | + |
| 72 | F | L | ++ | 0 | + |
| 68 | F | N | 0 | 0 | + |
| 66 | F | L | + | + | 0 |
| 70 | F | D | + | + | 0 |
| 70 | F | L | ++ | ++ | + |
| 67 | F | D | 0 | + | ++ |
| 77 | M | N | ++ | ++ | + |
| 76 | F | N | + | + | 0 |
| 72 | F | L | + | 0 | 0 |
| 31 | F | L | + | 0 | 0 |
| 18 | M | D | 0 | 0 | 0 |
| 28 | F | N | + | 0 | 0 |
| 64 | M | N | + | 0 | 0 |
| 67 | M | D | + | ++ | 0 |
| 65 | F | L | ++ | + | ++ |
| 50 | F | N | ++ | + | ++ |
| 50 | F | N | ++ | 0 | ++ |
| 47 | F | N | 0 | 0 | ++ |
| 77 | F | L | ++ | + | + |
| 70 | F | L | ++ | 0 | + |
| 81 | F | L | ++ | + | ++ |
| 75 | M | L | ++ | ++ | ++ |
| 70 | F | L | ++ | ++ | ++ |
| 76 | F | L | ++ | 0 | ++ |
| 73 | M | L | ++ | ++ | ++ |
| 71 | F | N | + | ++ | + |
| 71 | F | L | ++ | 0 | 0 |
| 71 | F | N | ++ | + | ++ |
| 66 | M | L | ++ | 0 | ++ |
| 15 | F | L | + | 0 | 0 |
| 80 | F | L | + | + | + |
| 56 | F | L | 0 | 0 | 0 |
